# Effect of calcination on coarse gangue rejection of hard rock lithium ores

**DOI:** 10.1038/s41598-022-17277-x

**Published:** 2022-07-28

**Authors:** Muhammad Kashif Nazir, Laurence Dyer, Bogale Tadesse, Boris Albijanic, Nadia Kashif

**Affiliations:** Western Australia School of Mines, Curtin University, Kalgoorlie, WA 6430 Australia

**Keywords:** Engineering, Chemical engineering

## Abstract

Processing of spodumene ores requires calcination as a compulsory pre-treatment to convert α-spodumene to a more reactive β-spodumene phase. This transformation takes place at an elevated temperature of above 900 °C and results in a 30% volumetric expansion of the mineral and the product having highly altered physical properties. This work examines these induced properties and the effect of calcination on lithium grade deportment with particle size. XRD analysis showed a significant amount of β-spodumene in the calcined finest fraction (i.e. the particles less than 0.6 mm). A marked reduction in the bond ball mill work index of the calcined lithium samples (i.e. 42.3%) was recorded supporting the observed fracturing and friable appearance of the sample following α to β-spodumene conversion. The deportment of lithium to finer fractions was significantly increased when the sample was calcined, indicating selective breakage of the spodumene over gangue minerals.

## Introduction

The application of lithium compounds in the battery industry has increased worldwide lithium demand. Of the two important resources of lithium, extraction from brines is commercially more viable as compared with hard rock mining. However, attention has shifted to an extent towards lithium extraction from more evenly distributed hard rock ores because of two reasons. Firstly, this commodity is monopolised due to its presence in some specific regions. Secondly, the compound annual growth rate of lithium is expected to be 25.5% that is a rise from 47.3 to 117.4 kt of lithium over 4 years between 2020 to 2024 forcing the expansion of production into other feedstocks^1^. This growth is mainly the result of increased sales of electric vehicles which are projected to rise from 3.4 million vehicles in 2020 to 12.7 million by 2024^1^.

Spodumene (LiAlSi_2_O_6_) is the most economically exploitable lithium-bearing mineral and is widely used in lithium extraction^2^. It is found in pegmatites and may occur with other lithium-bearing compounds such as petalite (LiAlSi_4_O_10_) and lepidolite (K(Li,Al,Rb)_3_ (Al,Si)_4_O_10_ (F,OH)_2_). The processing of lithium ores starts with beneficiation such as gravity separation/dense media separation, magnetic separation and/or flotation^3^. Beneficiation of lithium from spodumene is not a simple process because of similar properties of lithium-bearing minerals (i.e. spodumene) and their associated gangue minerals i.e. quartz (SiO_2_), albite (NaAlSi_3_O_8_), microcline (KAlSi_3_O_8_) and muscovite (KAl_2_(Si_3_Al)O_10_(OH,F)_2_).

A spodumene concentrate containing more than 6% Li_2_O is considered high-grade^4^ corresponding to at least 75% spodumene. This concentrate is suitable to feed the next processing stages: heat treatment and lithium extraction^2^. Heat treatment is required due to the natural existence of spodumene in a less reactive α-phase^5^. Heat treatment at elevated temperatures (above 950 °C) is an important step in lithium production because this step transforms less reactive α-spodumene into more reactive β-spodumene^6^. This phenomenon of phase transformation is called calcination or decrepitation which is an endothermic reaction^7^.

α-Spodumene is the most dominant naturally occurring of the three possible phases of spodumene, namely: α, β, γ. α-Spodumene (monoclinic crystal structure) is a denser and less reactive phase found at ambient temperature. However, β-spodumene (tetragonal crystal structure) is 30% less densely packed than α-spodumene. Thus, β-spodumene has lower specific gravity than α-spodumene (2.4 and 3.15 g/cm^3^ respectively). Hexagonal γ-spodumene is recently discovered and is metastable because it is formed during the transition from α to β phase^8^.

α-Spodumene presents as a competent rock, while β-spodumene is much more brittle than primary gangue minerals in the ore (eg, quartz)^7^. A microscopic study reveals α-spodumene as a compact material composed of multiple layers stacked over each other. Conversely, in β-spodumene samples, many cracks can be observed on particles leading to a more random crystal structure.

Based on the significant and potentially selective change in physical properties of spodumene the objective of this work is to investigate the implications of calcination on behaviour of the samples during comminution and grade deportment by size (coarse gangue rejection). In this work, different comminution techniques were used for calcined spodumene samples: crushing, semi-autogenous grinding, and autogenous grinding. The reason is that different comminution techniques result in varied particle size distributions and hence different grade deportment by size. In other words, crushing produces the coarsest fractions while semi-autogenous grinding generates the finest fractions. It should be noted that although it is understood that calcination of the whole ore would involve a marked increase in energy usage, the potential for separation and upgrading for challenging ores or mineralised waste streams is of interest.

## Materials and methods

### Samples

Spodumene ores were provided from a mine in the Eastern Goldfields, Western Australia. The received ores had particle size −7 mm i.e. the particles less than 7 mm. The ore sample was collected by belt cut from the final crusher product and is designated as the plant feed (PF). The other sample was collected by strafing a bucket through the tailings stream of the secondary (cleaner) dense media separation (TDMS). Table 1 shows the mineralogy of PF and TDMS samples. The lithium contents in the TDMS and PF samples were 0.36% and 0.20%, respectively. The lithium content was determined by forming a borate glass fusion bead, digestion in 10% citric acid, and subsequent ICP-OES (Agilent Technologies, Inc., USA), analysis. The solution was transported by a peristaltic pump in the nebulizer to convert the solution into a fine aerosol spray. Finer droplets enter the hot plasma, leading to evaporation of the sample. As a result, atoms and ions are excited causing the characteristic wavelengths emission which is quantified by the ICP-OES system; the wavelength used for ICP-OES analysis for lithium^9^ was 610.4 nm.

**Table 1:** Mineralogy of the spodumene ore.

Mineral	% Mass	
	TDMS	PF
Spodumene	10.6	8.3
Feldspar	70.2	71.5
Quartz	13.4	13.9
Mica	5.5	5.9
Others	0.3	0.4

### Calcination

The ore samples were calcined for 1 h at 1100 °C in a muffle furnace (Cupellation furnace, Carbolite Sheffield England). The holding time of one hour allowed complete conversion of spodumene from α-phase to β-phase for accurate results. The calcined and non-calcined samples were used to understand the influence of calcination on comminution operations (crushing, autogenous milling or semi-autogenous milling). The mill had a low-ball loading (10%) in contrast to standard ball milling (50%) and thus the mill was used to simulate a semi-autogenous grinding mill.

### Comminution

**Figure 1:**
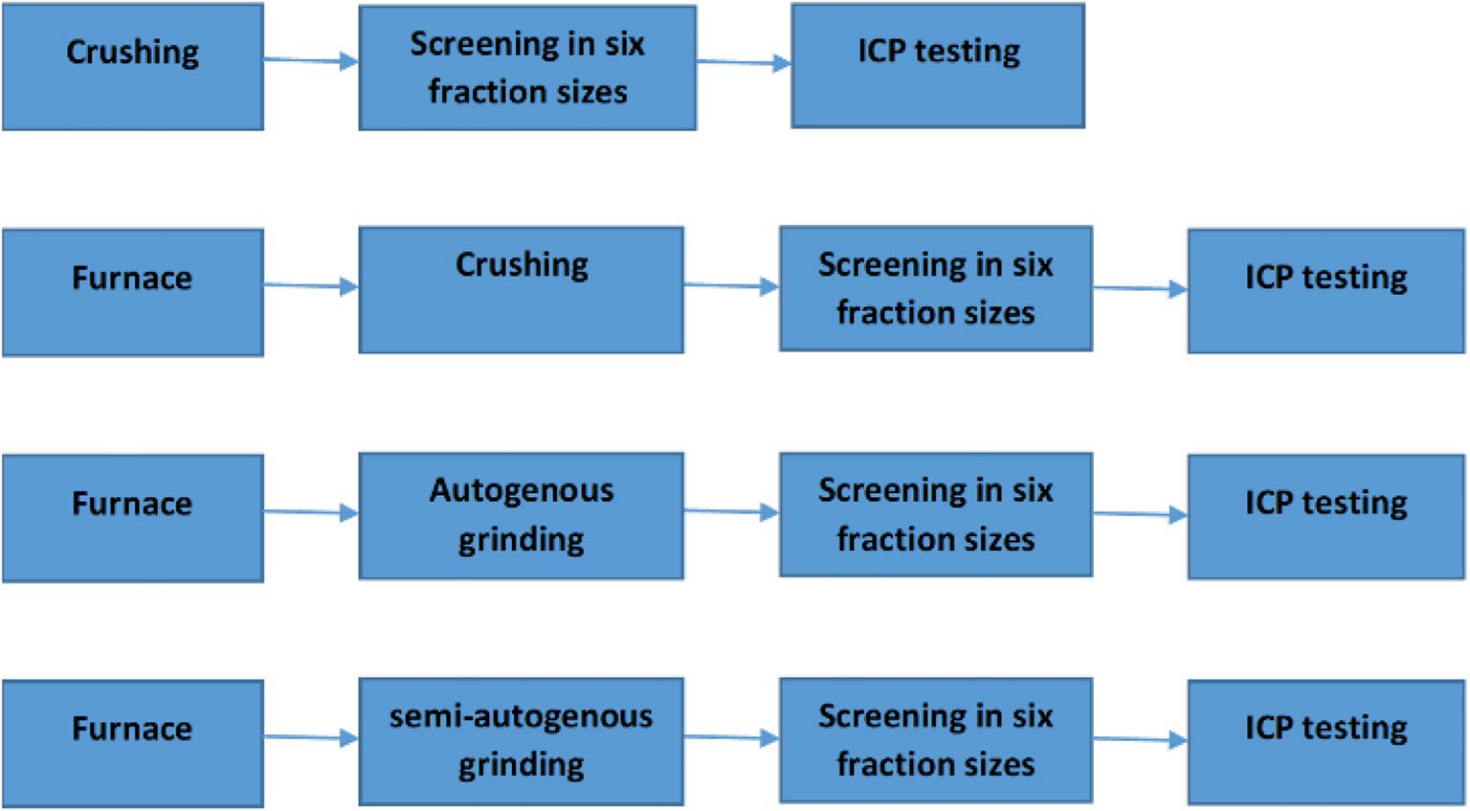
The experimental flowcharts.

Figure 1 shows the flowcharts used in this work. As seen in Fig. 1, underwent a range of comminution processes to provide information under a range of breakage processes (semi-autogenous or autogenous mill), screened in six different size fractions (+ 3.35 mm, −3.35 + 2.36 mm, −2.36 + 1.7 mm, −1.7 + 1.18 mm, −1.18 + 0.6 mm, −0.6 mm) and then the lithium grades were determined using ICP-OES (Agilent Technologies, US) in each size fraction; the standard deviation for three repeats of the lithium grade did not exceed 3%. The lithium recoveries (R) were determined using Eq. (1):

1 $$R=\frac{{m}_{p}\times {g}_{p}}{{m}_{f}\times {g}_{f}}$$

where ${m}_{p}  \, \mathrm{and } \, {m}_{f}$ are the mass of the product and the feed, respectively; ${g}_{p}$ and ${g}_{f}$ are the lithium grade in the product and the feed.

Crushing was performed using a cone crusher (Wescone, Australia) with a motor power of 9.2 kW; the closed side setting of the crusher is 3 mm. Semiautogenous and autogenous grinding were performed using a mill (the motor power of 1 kW) for 20 min. Semiautogenous grinding was conducted using 12 grinding balls (each grinding ball had 27.3 mm in diameter) that had a total mass of 1060 g; the rotational speed of the mill was 70 rpm. The calcined ores were investigated using crushing, semi-autogenous or autogenous mill.

### Bond ball mill work index

The Bond Ball Mill Work Index (BBMWI) is defined as the resistance offered by a material to the grinding during ball milling^10^. The purpose is to find out the grinding power required for a certain throughput of material under ball mill grinding circumstances. Different steel balls were used for grinding in each test as seen in Table 2. BBMWI was determined for both non-calcined and calcined samples. The standard bond ball mill procedure was followed^10^.

**Table 2:** Grinding balls used for BBMWI.

Ball diameter (mm)	Number of balls
38.10	43
31.75	67
25.40	10
19.05	71
15.87	94
Mass of balls	20.125 kg

The power required for grinding, Wi, was determined using Eq. (2)^10^.

2 $${W}_{i}=\frac{44.5\times 1.102}{{S}^{0.23}\times {G}^{0.82}\left(\frac{10}{\sqrt{{P}_{80}}}-\frac{10}{\sqrt{{F}_{80}}}\right)}$$

where the screen size required for 80% of a product or a feed to pass through the screen are P_80_ and F_80_, respectively; F_80_ and P_80_ were 1700 µm and 53 µm, respectively. G is the ore grindability and S is the sieve size through which ore passes.

### X-ray diffraction

Mineralogical analyses of the lithium ore samples were conducted using an Olympus BTX™ II Benchtop (Co-Kα) X-ray diffractometer (XRD). The XRD experiments were performed using two calcined finest fractions (−0.6 mm) and two non-calcined coarsest fractions (+ 3.35 mm) considering that these samples had the maximum lithium content. This is very important to identify changes in the crystalline structure of spodumene before and after calcination.

## Results and discussions

### Effect of calcination and comminution method on mass retention

**Figure 2:**
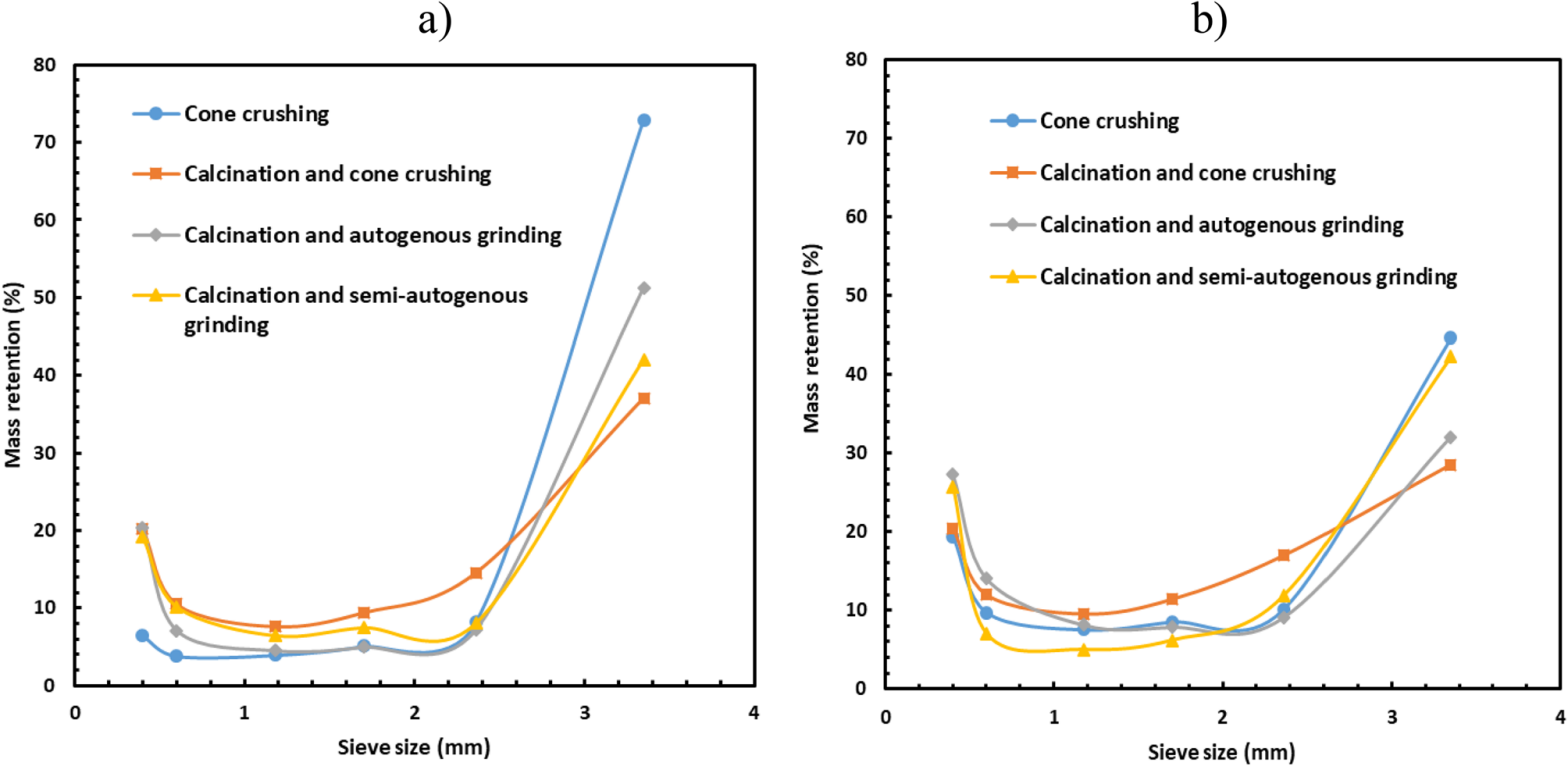
Retention of (**a**) TDMS, (**b**) PF on different sieves.

Figure 2 shows the influence of calcination and comminution method on mass retention on different sieve sizes for the TDMS and the PF sample. As seen in Fig. 2a., in the case of the TDMS sample, the highest percentage of ore was found on the largest size fraction when the ore was treated using the crushing only. It means that ore particles have high hardness considering that spodumene in the ore is in α-phase at 25 °C and has a compact crystal structure^2,7^. However, when calcination was performed before any comminution methods, the mass retention of the largest size fraction decreased significantly inducing fracturing before crushing. The difference between the non-calcined and calcined material is greater with more communition.

Figure 2 also shows that the lowest mass retention for the largest size fraction was when the ore was calcined followed by cone crushing. Similar trends were also obtained in the case of the PF sample. However, in the case of the PF (Fig. 2b), calcination followed by semi-autogenous grinding had more mass retention of the largest size fraction than calcination followed by autogenous grinding. The smallest mass in the largest size fraction was obtained after calcination and cone crushing; the reason could be that the calcined ore was more brittle and thus more easily crushed than treated by autogenous grinding or semi-autogenous grinding.

### Effect of calcination and comminution method on lithium grade and lithium recovery

**Figure 3:**
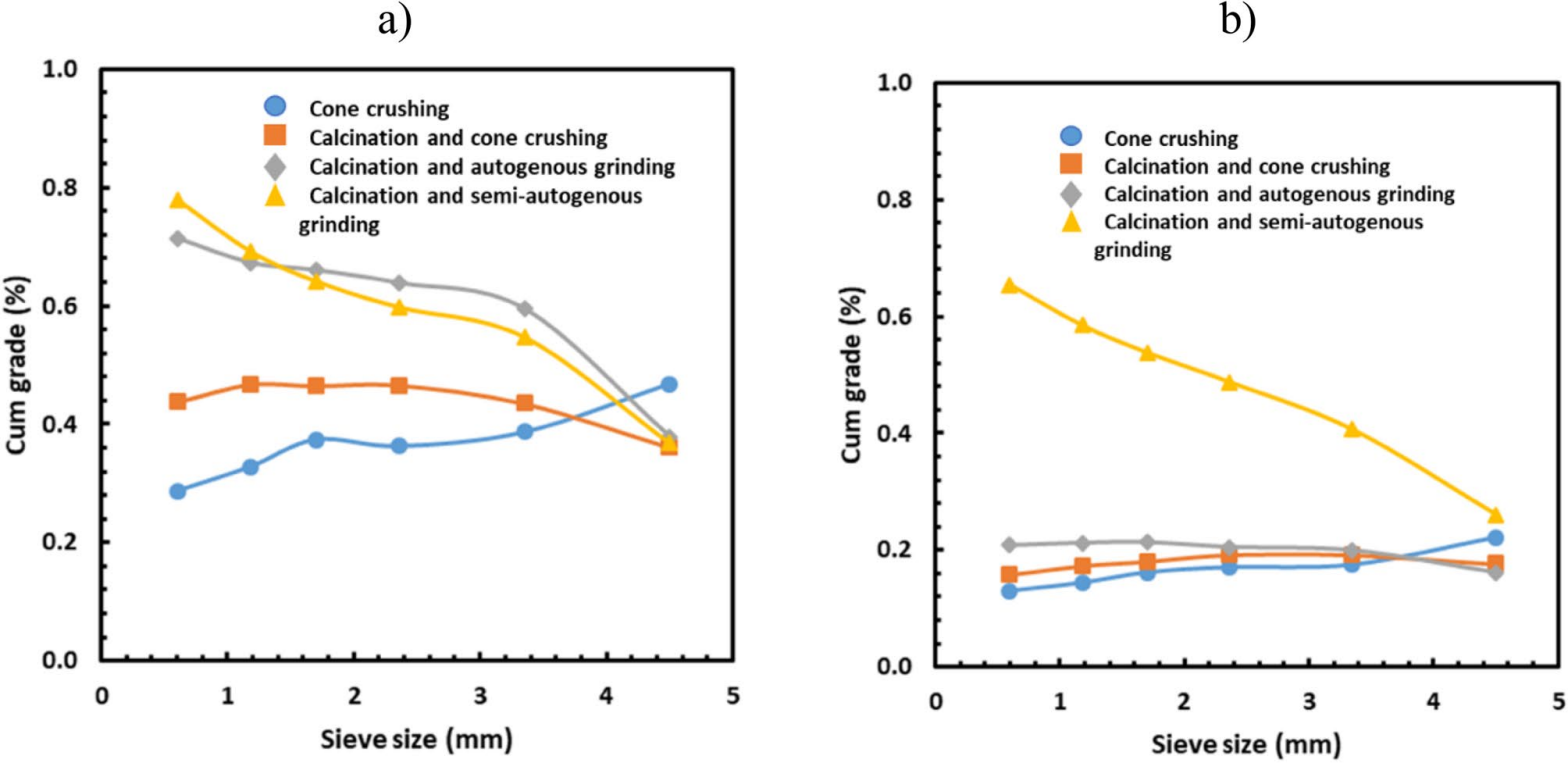
Influence of calcination and comminution methods on cumulative grade of lithium in the case of (**a**) TDMS and (**b**) PF.

Figure 3 shows the influence of the calcination and comminution method on the lithium grade for various size fractions for the TDMS and the PF sample. In the case of the TDMS sample (Fig. 3a), the cone crushing method without calcination resulted in the maximum lithium grade of the largest size fraction while the lithium grade was lowest for the smallest size fraction. However, the opposite trend was observed when calcination was used before comminution. This is particularly true when autogenous grinding or semi-autogenous grinding was conducted after calcination i.e. the lithium grade for the finest size fraction was the highest, displaying the highest potential gangue rejection.

The calcination impact on coarse gangue rejection in the case of the PF sample (Fig. 3b) was similar to that in the case of the TDMS (Fig. 3a). However, the lithium grade in the finest fraction was significantly higher when semi-autogenous grinding was conducted after calcination than that when cone crushing and autogenous grinding were performed after calcination.

**Figure 4:**
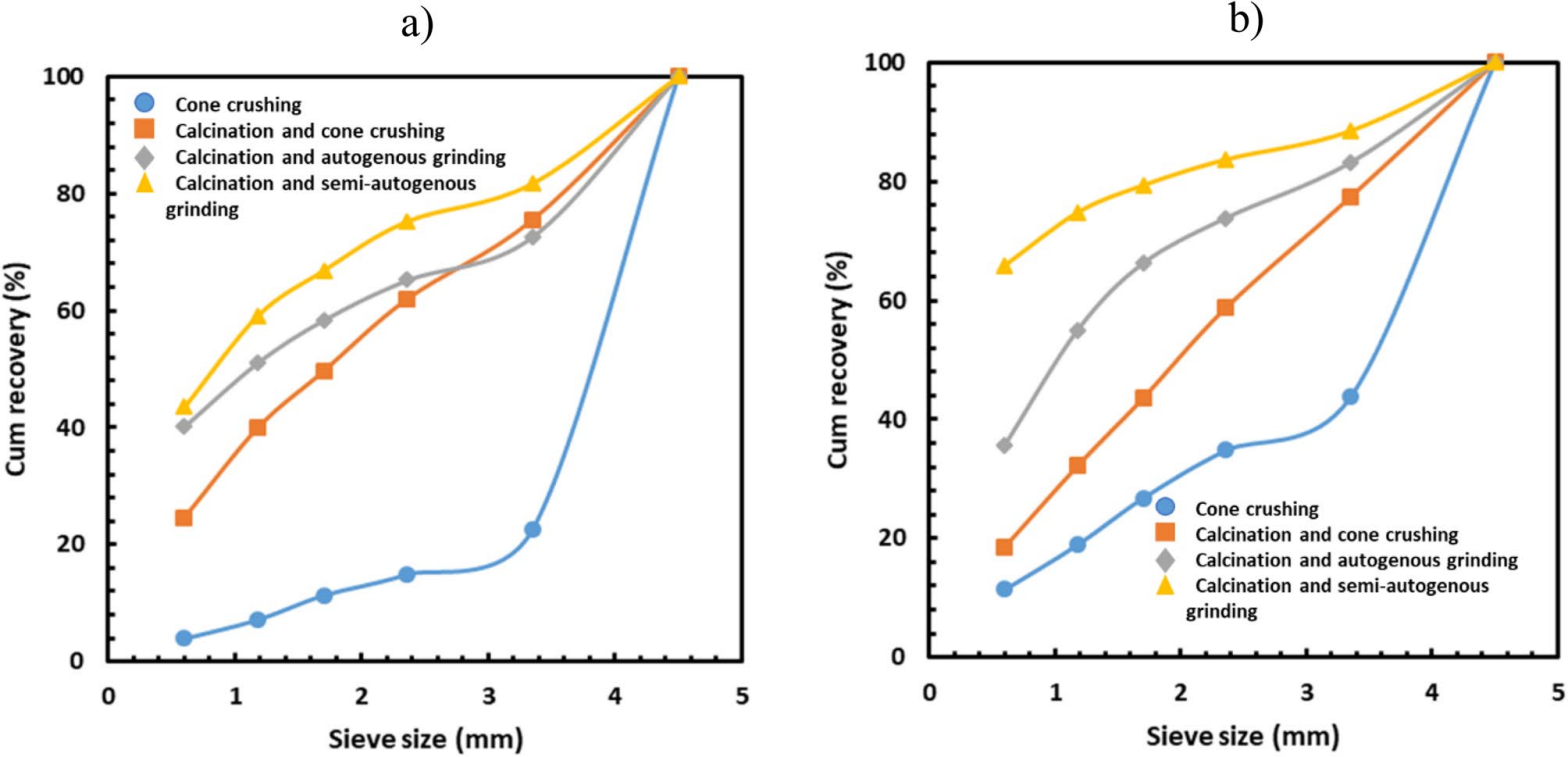
Influence of calcination and comminution methods on cumulative lithium recovery in the case of (**a**) TDMS and (**b**) PF.

The influence of calcination on lithium recovery can be seen in Fig. 4. As seen in Fig. 4, the lithium recovery after cone crushing and without calcination was the lowest at the finest screen size. However, when calcination was performed before crushing or grinding operations, the lithium recovery improved in the finest size fractions, leading to coarse gangue rejection. The highest lithium recovery was achieved when calcination was performed before semi-autogenous grinding.

**Figure 5:**
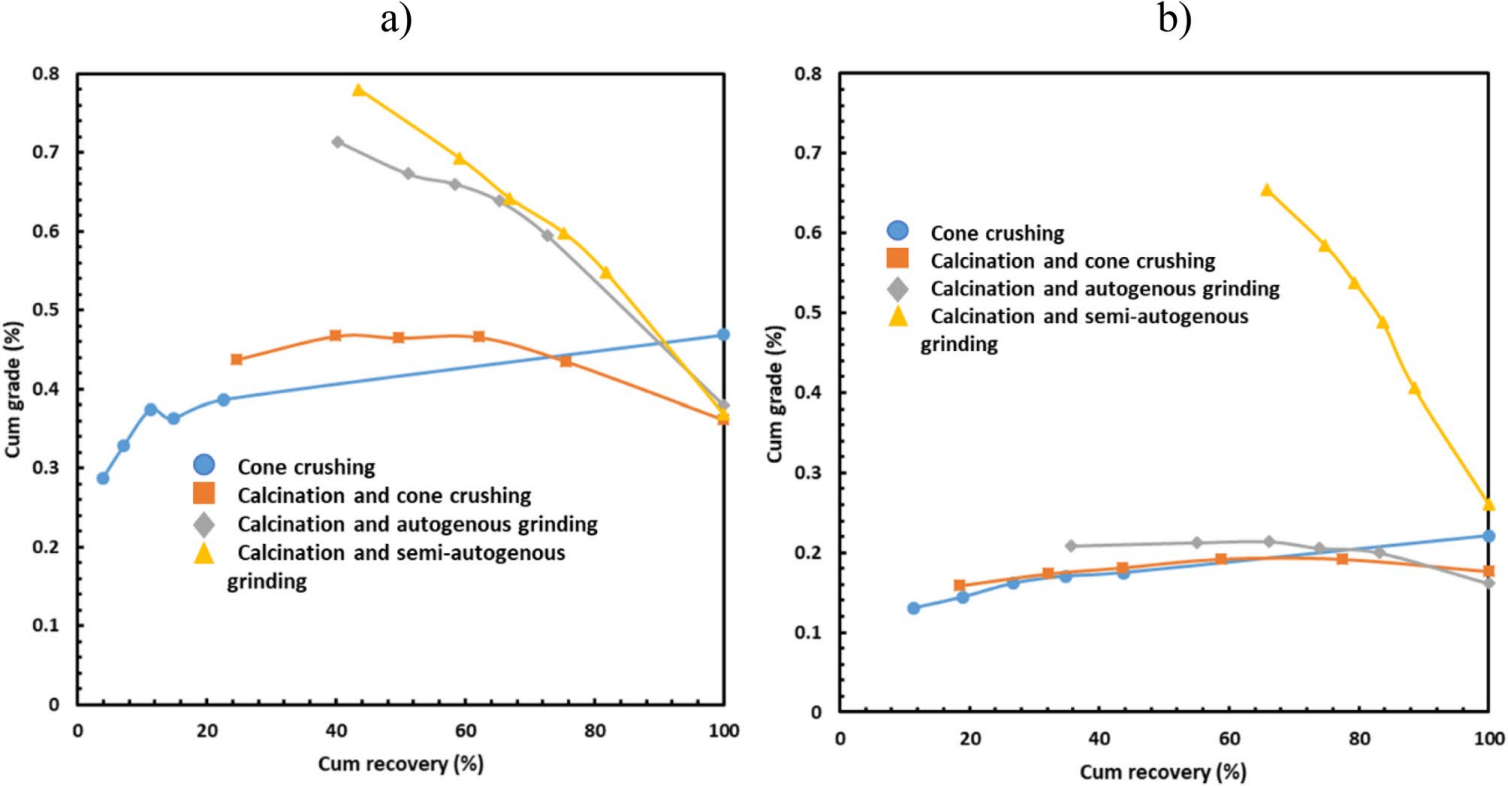
Cumulative grade vs cumulative recovery of lithium in the case of (**a**) TDMS and (**b**) PF.

Figure 5 shows the relationships between the cumulative grade and the cumulative recovery. The higher the lithium recovery the lower the lithium grade, which is true when calcination was used for both the TDSM (Fig. 5a) and the PF sample (Fig. 5b). It means that calcination may be effective in the rejection of coarse gangue. The effect of calcination was more pronounced when the samples were ground after calcination. However, in the case of the non-calcined sample, there was a preferential department of lithium to the largest size fraction because spodumene was present as a competent α phase.

**Figure 6:**
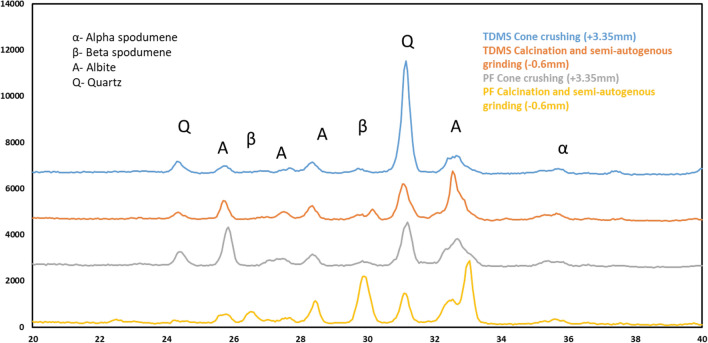
XRD for two calcined finest fractions (−0.6 mm) and two non-calcined coarsest fractions (+ 3.35 mm).

In the case of the TDMS sample, there are similarities between the grade recovery curves when calcination was performed before autogenous or semi-autogenous grinding; it may suggest that the TDMS sample was more amenable to autogenous grinding than the PF sample. In the case of the PF sample, the similarity between these curves was observed for the samples treated by cone crushing only, calcination and cone crushing, and calcination and autogenous grinding. The differences between the TDMS and the PF sample were probably due to their differences in gangue mineralogy (see Fig. 6). Even though the lithium content was low in both TDMS and PF, significant coarse gangue rejections were achieved only when calcination was performed before semi-autogenous grinding (i.e. the most efficient comminution method).

X-ray diffraction was used to investigate the impact on the mineral components of the samples through the various treatment schemes; Fig. 6 shows the diffraction patterns of the size fraction with the highest Li content from four sample sets. As anticipated β-spodumene replaces α-spodumene with calcination and is more prominent for both TDMS and PF samples as it is concentrated in the finer fraction^11,12^. For both TDMS and PF samples, the amount of albite also increased in the finest fraction of the calcined sample due to the albite transformation from the triclinic to monoclinic crystal structure^13^ and thus degradation in strength. Figure 6 also shows that the amount of quartz was higher in the non-calcined TDSM sample than that in the calcined TDMS sample showing that quartz was retained in the highest sieve size as it remained competent through calcination.

### Effect of calcination on lithium grade and recovery for very fine sizes

**Figure 7:**
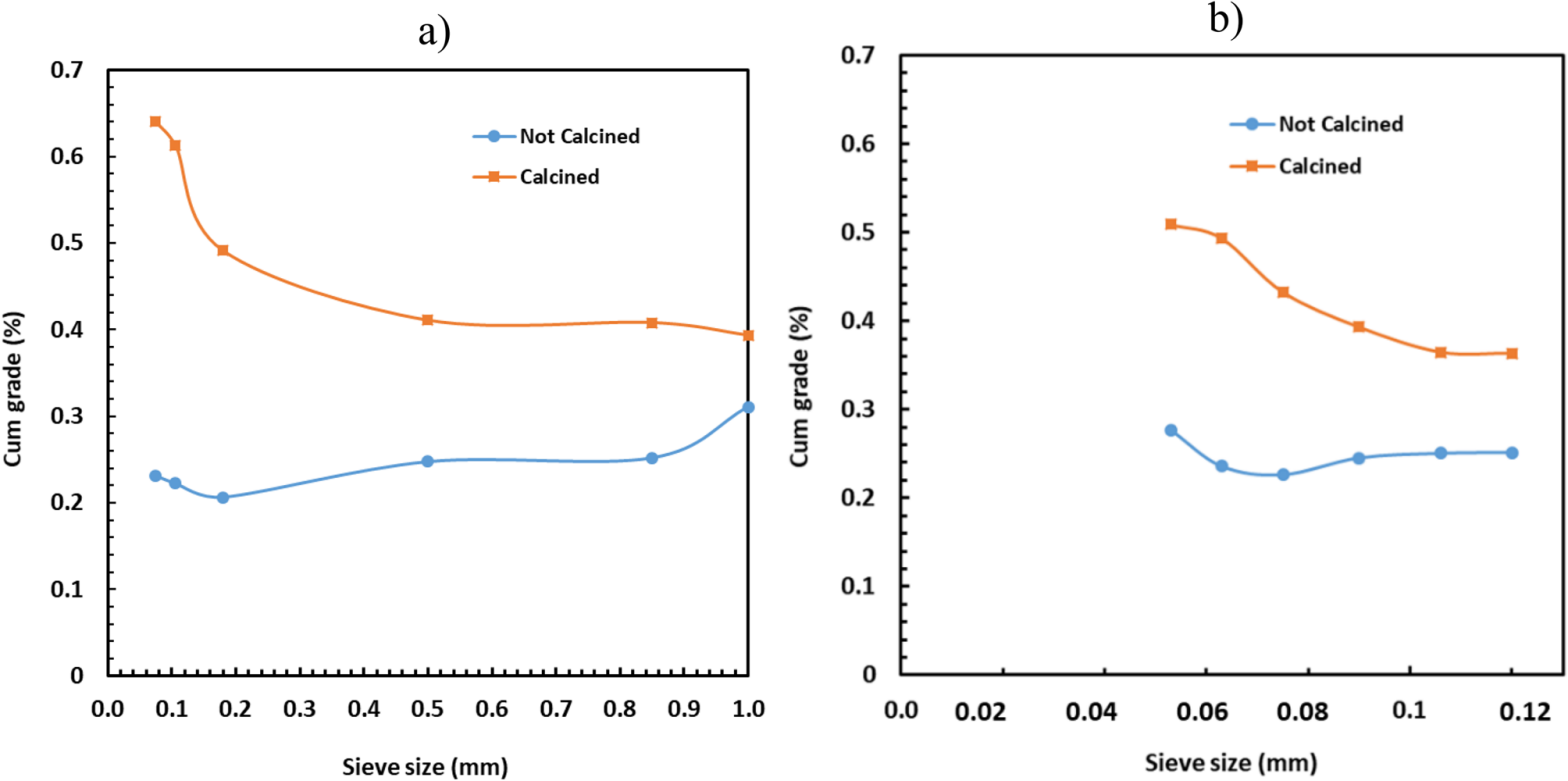
Influence of calcination on the cumulative grade of lithium in the case of (**a**) feed of the ball mill and (**b**) product of the ball mill.

**Figure 8:**
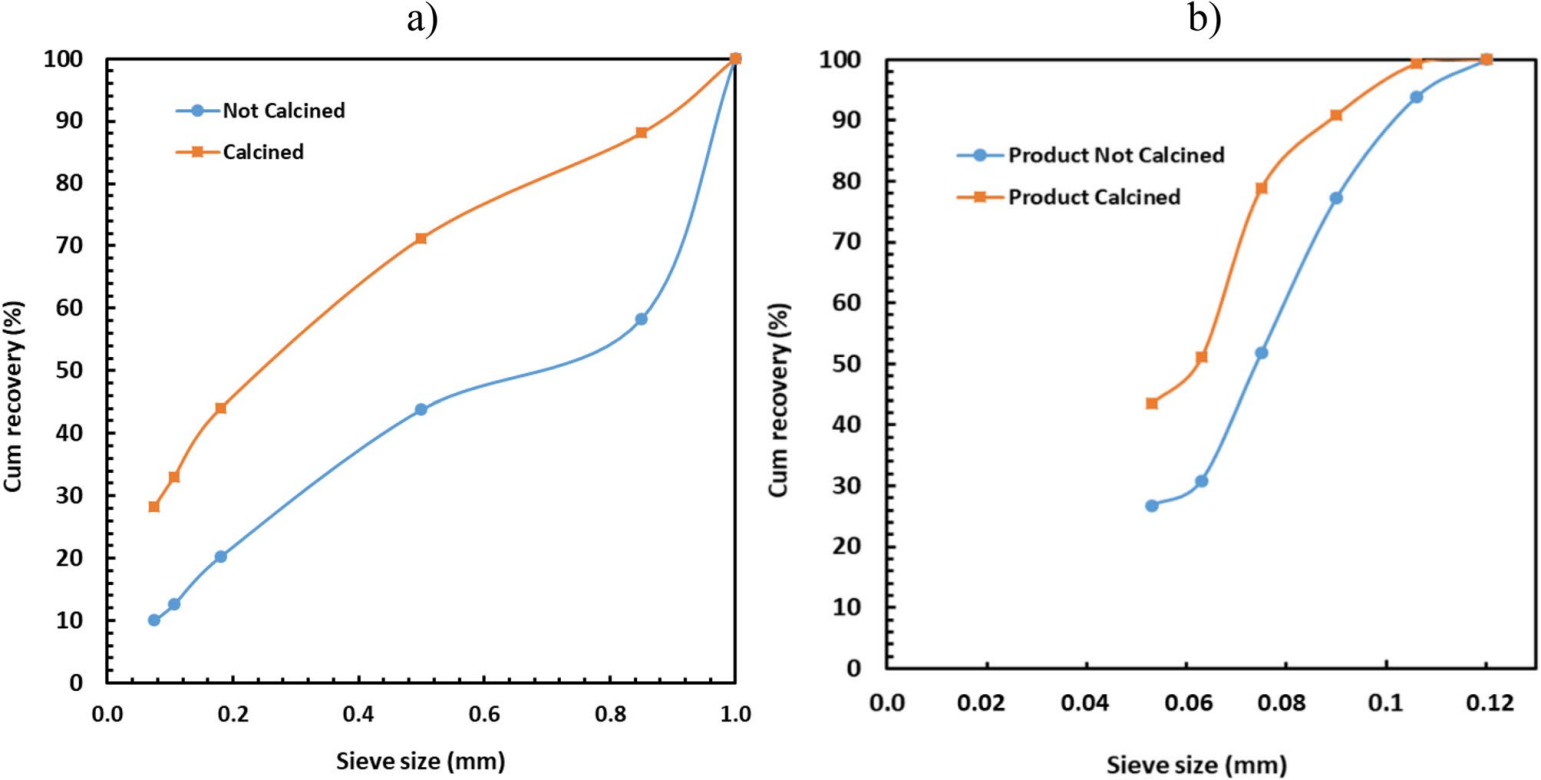
Influence of calcination on cumulative recovery of lithium in the case of (**a**) feed of BBMWI and (**b**) product of BBMWI in the case of PF.

The influence of calcination on lithium deportment to very fine sizes was investigated using the sieves in the range of 150 and 53 µm. Both feed and products from the ball mill were analysed to address this matter as seen in Figs. 7 and 8; the ball mill product was collected after conducting the BBMWI test. It was found that the calcination was also very beneficial for lithium grade by sieve size even at very fine sizes (150 and 53 µm). The increase in sieve size reduced the amount of lithium grade for the sample retained on the sieve but increased the cumulative lithium recovery. These trends were also observed in the case of the largest sieve sizes (see Figs. 3, 4, 5). However, Figs. 7 and 8 showed that the cumulative recovery increased more dramatically with increasing in sieve size in the case of the ball mill product than that of the feed.

### Effect of calcination on energy consumption during grinding

The results showed that the non-calcined ore required 1.73 times more energy for grinding than the calcined ore i.e. W_i_ (i.e. BBMWI) for the non-calcined ore was 44.9 kWh/t and for the calcined ore was 25.9 kWh/t. It should be noted that the non-calcined ore had significantly higher BBMWI than α-spodumene^14^ i.e. 13.70 kWh/t; the reason is due to the presence of different gangue minerals such as mica, quartz, albite and other silicates; the BBMWI for mica^14^ is 134.5 kWh/t, quartz^14^ is 32.2 kWh/t and albite^14^ is 34.9 kWh/t.

The energy consumed, *Q*, during calcinations of spodumene is obtained using the energy balance i.e. Equation (3):

3 $$\mathrm{Q}=\frac{m}{M}\underset{298}{\overset{1373}{\int }}{C}_{p}(T)dT+{\mathrm{Q}}_{\alpha \beta }$$

where ${C}_{p}(T)=$ 354.7–3375.7 T^−0.5^ J mol^−1^ K^−1^ as reported by Dessmond and colleagues^7^; *T* is the temperature; *m* is the mass of the sample; *M* is the molecular mass of spodumene (186 g/mol). It should be noted that the energy transformation from α to β spodumene, ${\mathrm{Q}}_{\alpha \beta },$ was 116.1 kJ/kg as reported by Dessmond and colleagues^7^. The energy consumed during calcination was 582 kWh/t or 2096 kJ/kg. Therefore, calcination before grinding resulted in increased overall energy consumption.

The summary of energy consumption for all unit operations is given in Table 3. The energy for crushing, autogenous grinding and semi-autogenous grinding were determined by considering the duration of each operation, motor power and sample mass. As seen in Table 3, the furnace consumes vastly more energy than the comminution circuit.

**Table 3:** Energy consumption for different unit operations.

Unit operation	Energy consumption (kJ/kg)
Furnace	2096
Crusher	69
Autogenous grinding	75
Semiautogenous grinding	237

Table 4 compares energy consumption and lithium grade for the finest size fractions (−0.6 mm) for four different processing options used in this work. As seen in Table 4, although the combination of the furnace with the crusher or the mill (autogenous grinding or semi-autogenous grinding) increased the energy consumption of the process, using the furnace increased the lithium grade by the screening of hard rock lithium ores. It is also very important to highlight that if calcination is not used before grinding, calcination is used always after grinding and before leaching since leaching of spodumene is not possible without calcination. When ore calcination is conducted before leaching or after flotation, the amount of energy consumed for calcination is approximately 1257.6 kJ/kg considering that flotation concentrates have typically 60% of spodumene^3^ (i.e. 2096 × 60/100 = 1257.6 kJ/kg; 2096 kJ/kg is the energy consumption of calcination for pure spodumene as seen in Table 3). It is important to highlight that the main objective of this paper is not to develop a new flowsheet but to investigate the implications of calcination on behaviour of the samples during comminution and grade deportment by size (coarse gangue rejection).

**Table 4:** Energy consumptions and lithium grade for −0.6 mm for different processing operations.

Processing options	Energy consumption (kJ/kg)	Li grade % (TDSM) (−0.6 mm)	Li grade % (PF) (−0.6 mm)
Crusher	69	0.28	0.12
Furnace + crusher	2165	0.44	0.16
Furnace + mill (autogenous grinding)	2171	0.72	0.2
Furnace + mill (semiautogenous grinding)	2333	0.76	0.64

## Conclusions

This paper studies the influence of calcination of spodumene ore and comminution circuits on coarse gangue rejections by screening. The results showed that the calcination made spodumene brittle, having a positive effect on coarse gangue rejection by increasing lithium grade and recovery in the finest fraction. This effect was observed when the sieve size was in the range between 0.6 and 5 mm as well as 0.063 and 1 mm. The results of this work display the significantly altered properties of calcined material that promote preferential breakage of the spodumene over other components. Semi-autogenous grinding after calcination generated significantly more fines than autogenous grinding or crushing after calcination in the case of the PF sample. The energy consumed during the bond ball mill work test of the calcined ores was 42% less than that of the non-calcined ores. It must be noted that the reduction in comminution energy does not account for the additional energy consumption in calcining feed streams rather than concentrates.

## References

[1] Fawthrop, A. *Global Lithium Demand to More Than Double by 2024, Say Analysts*. https://www.nsenergybusiness.com/news/industry-news/global-lithium-demand-2024/. Accessed 16 Dec 2021 (2020).

[2] Peltosaari O, Tanskanen PA, Heikkinen EP, Fabritius T (2015). α→γ→β-phase transformation of spodumene with hybrid microwave and conventional furnaces. Miner. Eng..

[3] Tadesse B, Makuei F, Albijanic B, Dyer L (2019). The beneficiation of lithium minerals from hard rock ores: A review. Miner. Eng..

[4] Peltosaari O, Tanskanen PA, Heikkinen EP, Fabritius T (2016). Mechanical enrichment of converted spodumene by selective sieving. Miner. Eng..

[5] Rosales GD, Ruiz MDC, Rodriguez MH (2014). Novel process for the extraction of lithium from b-spodumene by leaching with HF. Hydrometallurgy.

[6] Salakjani NK, Singh P, Nikoloski AN (2016). Mineralogical transformations of spodumene concentrate from Greenbushes, Western Australia. Part 1: Conventional heating. Miner. Eng..

[7] Dessemond, C., Lajoie-Leroux, F., Soucy, G., Laroche, N. & Magnan, J.-F. *Spodumene: The Lithium Market, Resources and Processes, Minerals*. Accessed 12 Mar 2021. (2019).

[8] Li CT, Peacor DR (1968). The crystal structure of LiAlSi2O6-II (β-spodumene). Z. Kristallogr..

[9] Bach TC, Schuster SF, Fleder E, Muller J, Brand MJ, Lorrmann H, Jossen A, Sextl G (2016). Nonlinear aging of cylindrical lithium-ion cells linked to heterogeneous compression. J. Energy Storage.

[10] Lynch, A., Mainza, A. & Morell, S., Ore comminution measurements techniques. in: *The AusIMM Comminution Handbook Carlton: AusIMM*. (Lynch, A.J. ed.). 43–60. (2015).

[11] Ellestad, R.B. & Leute, K.M. *Method of Extracting Lithium Values from Spodumene Ores. U.S. Patent 2516109*. (1950).

[12] Abdullah AA, Oskierskia HC, Altarawneh MN, Senanayake G, Lumpkin G, Dlugogorski BZ (2019). Phase transformation mechanism of spodumene during its calcination. Miner. Eng..

[13] Shaocheng JS, Mainprice D (1987). Experimental deformation of sintered albite above and below the order-disorder transition. Geodin. Acta.

[14] Perry, R. O. & Chilton, C. H. *Mineral Processing Handbook* (Int Student's ed., McGraw Hill. 8–11/SME, Weiss ed. 3A-27, 1985).

